# Chronic Uncontrolled Hypothyroidism Associated With Dysphonia and Concurrent Pericardial Effusion

**DOI:** 10.7759/cureus.25261

**Published:** 2022-05-23

**Authors:** Rishu Batra, Gordon Glober, Andrew Gonedes, Jay Patel, Esra Sari, Jessica El-Bahri

**Affiliations:** 1 Internal Medicine, Orange Park Medical Center, Orange Park, USA; 2 Internal Medicine, HCA Florida Orange Park Hospital, Orange Park, USA

**Keywords:** hypothyroid-related dysphonia, pericardial effusion, echocardiography, vocal fold edema, hypothyroid myxedema coma, hypothyroid pericardial effusion

## Abstract

Hypothyroidism is a commonly encountered pathology within internal medicine. It commonly presents with symptoms of fatigue, weight gain, constipation, and dry skin. Long-standing uncontrolled hypothyroidism can manifest with atypical symptoms of dysphonia and even pericardial effusion. This constellation of findings is not often encountered concurrently. While likely a consequence of uncontrolled hypothyroidism, it is prudent to ensure appropriate protection of the patient’s airway and rule out other obstructive causes of dysphonia, such as malignancy. We present the case of a patient with uncontrolled hypothyroidism who presented with dysphonia. While treating hypothyroidism, the patient was found to have pericardial effusion. Other causes of obstruction such as vocal cord dysfunction and malignancy were ruled out via imaging studies and multidisciplinary discussion with other subspecialties.

## Introduction

Hypothyroidism is a systemic disease characterized by dysregulation of thyroid hormone signaling that has myriad clinical manifestations. Broadly, hypothyroidism manifests clinically by the generalized slowing of metabolic processes, in addition to the accumulation of matrix glycosaminoglycans in the interstitium of many tissue types [1]. As metabolic processes slow, patients commonly demonstrate generalized symptoms such as fatigue, weakness, cold intolerance, and weight gain. Further, as glycosaminoglycans deposit diffusely, patients frequently demonstrate non-pitting edema of the extremities, periorbital region, and elsewhere throughout the body including the larynx, manifesting as dysphonia [2,3].

Hypothyroid states affect the physiology of the cardiac system and, in rare or severe cases, can lead to critical pathology [3,4]. These changes are mediated both directly and indirectly via thyroid hormone insufficiency. Inotropy and chronotropy of the heart decrease leading to bradycardia while vasculature becomes dysregulated. In severe cases, this may manifest as heart failure or cardiomyopathy [3], arrhythmias, diastolic hypertension [5], and atherosclerotic disease. Interestingly, in rare severe cases, pericardial effusion has been observed in patients and is thought to be due to increased capillary permeability [6]. Although typically indolent, if left untreated, it can potentially lead to cardiac tamponade [7].

## Case presentation

A 51-year-old Caucasian female with a medical history of papillary thyroid cancer status post-thyroidectomy and known hypothyroidism presented for evaluation after a motor vehicle accident. She was a restrained driver in a non-traumatic, low-impact collision with no deployment of airbags. On initial evaluation, she could not recall the details of the collision. She admitted to waxing and waning memory impairment, dysphonia, vision disturbances, and gross anasarca for the past several months. While she noted her home levothyroxine dose to be around 150 µg daily, the patient acknowledged she was not confident of the dosing and stated she was not regularly taking the medication. She stated that the dysphonia had gradually progressed, coinciding with her concurrent inconsistency with taking her thyroid hormone replacement. On admission, initial vital signs were stable. A physical examination showed gross anasarca, bilateral ptosis, and severe hoarseness. Her pertinent labs included a thyroid-stimulating hormone (TSH) level of 113.63 mU/L, with free thyroxine (T4) level of 0.17 pmol/L on the day of admission. An ultrasound of the neck confirmed no residual thyroid tissue and normal lymph nodes bilaterally.

**Figure 1:**
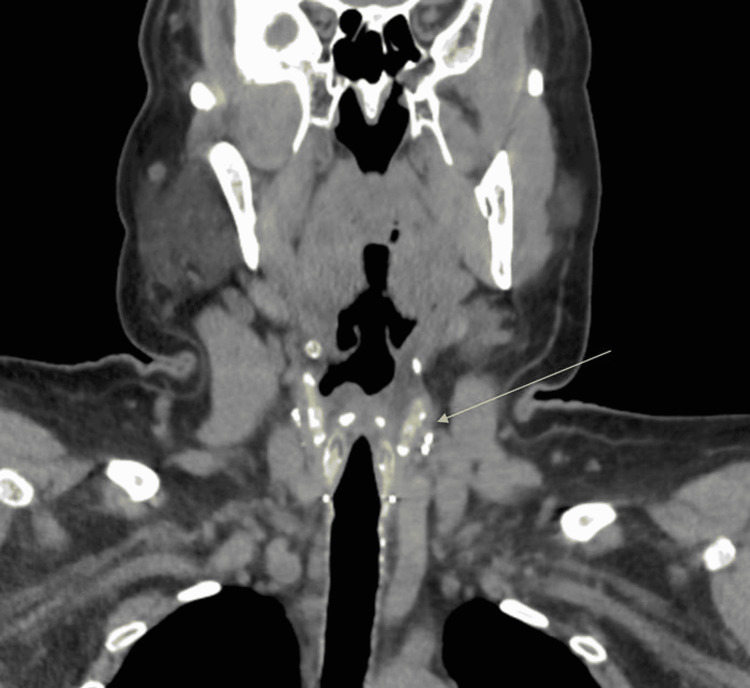
CT of the neck without contrast indicating post-treatment changes related to prior thyroidectomy, with no residual present thyroid tissue or any pathologically enlarged lymph nodes. Arrow pointing to the paratracheal surgical clips after prior thyroidectomy. CT: computerized tomography

**Figure 2:**
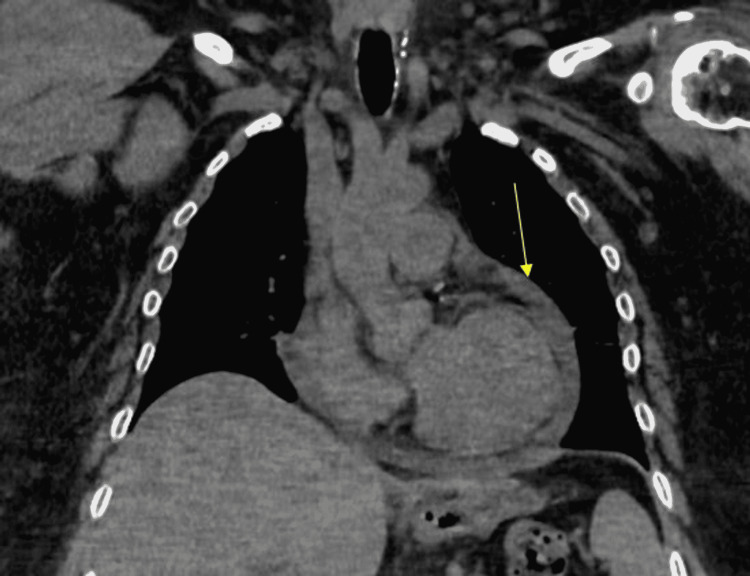
CT chest coronal reformatted image without contrast demonstrating small-to-moderate-sized pericardial effusion. Arrow pointing to pericardial effusion. CT: computerized tomography

The patient’s oral levothyroxine dose was re-calculated and restarted on admission to 200 µg daily intravenously (IV). Computerized tomography (CT) of the head and neck again confirmed the presence of no residual thyroid tissue (Figure 1). Otolaryngology (ENT) was consulted to further investigate any possible functional or anatomic etiology of persistent hoarseness. The patient underwent a laryngoscopy which only showed reflux-related changes but normal anatomy and vocal fold movement. To appropriately rule out other pathologies, gastroenterology (GI) proceeded with an endoscopy with biopsy to rule out uncontrolled gastroesophageal reflux (GERD) or obstruction as the etiology of dysphonia. She was found to have Los Angeles (LA) Grade B esophagitis, recent stigmata of bleeding from gastric erosions, and a small hiatal hernia. A biopsy was negative for *Helicobacter pylori*. The patient was started on pantoprazole 40 mg daily. It was decided to obtain a CT of the chest, which showed a small-to-moderate pericardial effusion (Figure 2). Cardiology proceeded with a transthoracic echocardiogram which indicated small-sized, chronic pericardial effusion with no evidence of hemodynamic compromise. After multidisciplinary discussion with ENT, GI, and endocrinology specialists, it was determined that the patient’s presenting symptoms were likely a result of longstanding uncontrolled hypothyroidism. The patient was continued on the 200 µg levothyroxine dose with notable improvement in hoarseness at discharge. The patient was advised to follow up with endocrinology as an outpatient for continual monitoring and management of her hypothyroidism.

## Discussion

Dysphonia in hypothyroidism is not an uncommon finding and can be due to multiple hypothyroid-mediated mechanisms [2]. The most direct cause of hypothyroid-induced dysphonia may be via myxedematous swelling of the vocal folds, known as Reinke’s edema, which leads to expected vocal fold dysfunction and resultant dysphonia [8]. In a more indirect fashion, hypothyroidism is known to predispose patients to GERD and reflux esophagitis due to gastrointestinal dysmotility, decreased stomach acid production paradoxically predisposing to reflux, and lower esophageal sphincter dysfunction [9].

In this case, we observed multiple pathways by which the patient’s hypothyroidism may have caused dysphonia. The patient’s bedside direct laryngoscopy indicated findings consistent with laryngopharyngeal reflux disease, a well-documented culprit of dysphonia. Importantly, no comment in the report was made on Reinke’s edema or gross vocal fold dysmotility. As such, we were unable to rule out these aforementioned etiologies with complete certainty. Additionally, we considered the possibility of left recurrent laryngeal nerve palsy via direct mechanical pressure by the patient’s enlarged pericardium. However, given no gross visualization of vocal fold dysmotility, ultimately we considered this possibility less likely ultimately.

We hypothesize that the cause of the dysphonia could potentially be multifactorial. Although uncontrolled acid reflux is known to cause voice disturbances, the timing and progression of the dysphonia correlated with the patient having missed follow-up with endocrinology and thereby not consistently taking her home thyroid replacement. While GERD can present silently, the patient overall denied symptoms of heartburn, chest pain, dysphagia, or any association with meals. With the patient’s rapid improvement with intravenous and subsequently oral levothyroxine, endocrinology believed that the underlying chronic hypothyroid played a larger role in the hoarseness. The patient was not started on acid suppression therapy till just prior to discharge and after improvement in symptoms.

The patient demonstrated hypothyroid-induced cardiac pathology showing a mild pericardial effusion on imaging via CT chest and echocardiography without signs of cardiac tamponade. This finding is consistent with observations of pericardial effusion in cases of severe hypothyroidism thought to be mediated by increased capillary permeability, which can ultimately lead to tamponade with obstructive shock physiology in extreme cases [4,5]. The patient’s echocardiography did not find evidence of cardiomyopathy or reduced ejection fraction, thus ruling out true hypothyroid-induced cardiomyopathy. However, the patient did demonstrate mild bradycardia, consistent with decreased chronotropic effect. The patient was started on appropriate thyroid hormone repletion, which in time would resolve the pericardial effusion, and no pericardiocentesis was warranted. Although recent trauma is a known cause of pericardial effusion, the motor vehicle accident was minor in nature, with the car traveling at standstill speeds causing no trauma and no deployment of airbags to the patient’s chest. The physical examination did not demonstrate any evidence of seat belt syndrome, and the patient did not endorse any acute symptoms after the car crash. This all lends credence to the noted pericardial effusion being more chronic in nature, and thereby more associated with hypothyroidism.

This case is a unique combination of manifestations of hypothyroidism end-organ dysfunction that have previously not been well-documented. Further, this case highlights some of the challenges of working up dysphonia in hypothyroidism. Especially in the setting of prior thyroid cancer, ruling out recurrence is vital to determine further course of management. While we do acknowledge there could be a multifactorial underlying etiology, with the patient’s findings being chronic in nature and noted swift improvement with appropriate thyroid replacement, hypothyroidism was thought to be the prime etiology. The patient’s presentation manifests the importance of medication compliance and the importance of consistent outpatient follow-up and monitoring.

## Conclusions

This case highlights the numerous ways in which hypothyroidism can present. Persistent dysphonia can be a difficult symptom to properly dissect. Oftentimes, there are no clear diagnostic clues as to the etiology of dysphonia. Judicial workup with multidisciplinary patient care is often warranted. The patient’s presentation manifests the importance of clinical acumen and relying on basic science principles for a diagnostic framework. We hope to bring greater awareness to dysphonia and pericardial effusion as adverse effects of longstanding, uncontrolled hypothyroidism, highlighting the importance of appropriate workup and treatment to prevent such adverse effects.
